# Towards Regional, Error-Bounded Landscape Carbon Storage Estimates for Data-Deficient Areas of the World

**DOI:** 10.1371/journal.pone.0044795

**Published:** 2012-09-14

**Authors:** Simon Willcock, Oliver L. Phillips, Philip J. Platts, Andrew Balmford, Neil D. Burgess, Jon C. Lovett, Antje Ahrends, Julian Bayliss, Nike Doggart, Kathryn Doody, Eibleis Fanning, Jonathan Green, Jaclyn Hall, Kim L. Howell, Rob Marchant, Andrew R. Marshall, Boniface Mbilinyi, Pantaleon K. T. Munishi, Nisha Owen, Ruth D. Swetnam, Elmer J. Topp-Jorgensen, Simon L. Lewis

**Affiliations:** 1 University of Leeds, Leeds, United Kingdom; 2 University of Cambridge, Cambridge, United Kingdom; 3 University of York, York, United Kingdom; 4 WWF US, Washington, D.C., United States of America; 5 University of Copenhagen, Copenhagen, Denmark; 6 University of Twente, Enschede, Netherlands; 7 Royal Botanic Garden, Edinburgh, United Kingdom; 8 Tanzanian Forest Conservation Group, Dar es Salaam, Tanzania; 9 Frankfurt Zoological Society, Frankfurt, Germany; 10 The Society for Environmental Exploration, London, United Kingdom; 11 University of Louvain-la-Neuve, Louvain-la-Neuve, Belgium; 12 The University of Dar es Salaam, Dar es Salaam, Tanzania; 13 Flamingo Land Ltd., Malton, United Kingdom; 14 Sokoine University of Agriculture, Morogoro, Tanzania; 15 University College London, London, United Kingdom; Ohio State University, United States of America

## Abstract

Monitoring landscape carbon storage is critical for supporting and validating climate change mitigation policies. These may be aimed at reducing deforestation and degradation, or increasing terrestrial carbon storage at local, regional and global levels. However, due to data-deficiencies, default global carbon storage values for given land cover types such as ‘lowland tropical forest’ are often used, termed ‘Tier 1 type’ analyses by the Intergovernmental Panel on Climate Change (IPCC). Such estimates may be erroneous when used at regional scales. Furthermore uncertainty assessments are rarely provided leading to estimates of land cover change carbon fluxes of unknown precision which may undermine efforts to properly evaluate land cover policies aimed at altering land cover dynamics. Here, we present a repeatable method to estimate carbon storage values and associated 95% confidence intervals (CI) for all five IPCC carbon pools (aboveground live carbon, litter, coarse woody debris, belowground live carbon and soil carbon) for data-deficient regions, using a combination of existing inventory data and systematic literature searches, weighted to ensure the final values are regionally specific. The method meets the IPCC ‘Tier 2’ reporting standard. We use this method to estimate carbon storage over an area of33.9 million hectares of eastern Tanzania, reporting values for 30 land cover types. We estimate that this area stored 6.33 (5.92–6.74) Pg C in the year 2000. Carbon storage estimates for the same study area extracted from five published Africa-wide or global studies show a mean carbon storage value of ∼50% of that reported using our regional values, with four of the five studies reporting lower carbon storage values. This suggests that carbon storage may have been underestimated for this region of Africa. Our study demonstrates the importance of obtaining regionally appropriate carbon storage estimates, and shows how such values can be produced for a relatively low investment.

## Introduction

Land cover change is known to make up a significant proportion of global greenhouse gas emissions. For example, anthropogenic destruction of tropical forests is responsible for between 10% to 28% of global carbon dioxide emissions, depending upon definitions [1]–[6]. In response to this, a broad agreement within the United Nations Framework Convention on Climate Change (UNFCCC) was reached to implement a scheme titled ‘Reducing Emissions from Deforestation and Forest Degradation’ (REDD) as a means to encourage the reduction of these emissions, later expanding the schemes' scope to include the sustainable management of forests and the conservation and enhancement of forest carbon stocks, termed REDD+ [7].

To have to opportunity to receive potential financial incentives through mitigation schemes such as REDD+, countries must estimate carbon storage and rates of loss, following guidance materials [8]–[10]. However, many developing countries lack the data to perform some of the recommended carbon accounting methods [7] and as such often resort to so-called ‘Tier 1’ analyses using global default carbon storage values for given land cover types [11], [12]. However, carbon stock is known to vary spatially on local [13] and global scales [14], [15]. Thus, regionally appropriate values, indicating uncertainties (‘Tier 2’), and those derived from intensive multiple census inventory data (‘Tier 3’) are preferable [12], [16]. This tiered approach has the advantage of enabling participation of all countries, despite varying data availability (Table 1).

**Table 1:** Description of the IPCC carbon pools and general tiers to estimate changes in carbon stocks in biomass in a land cover category, taken from [12].

IPCC term	Description
**Tier 1**	Uses aggregate data and default emission/removal factors
**Tier 2**	Uses country-specific biomass data and emission/removal factors
**Tier 3**	Uses detailed data on biomass to estimate changes in carbon stock using dynamic models or allometric equations
**Aboveground live carbon**	All carbon contained in living vegetation, both woody and herbaceous, above the soil including stems, stumps, branches, bark, seeds, and foliage.
**Coarse woody debris carbon**	All non-living woody carbon not contained in the litter, either standing, lying on the ground, or in the soil. Dead wood includes wood lying on the surface, dead roots, and stumps, larger than or equal to 10 cm in diameter (or the diameter specified by the country).
**Litter carbon**	All non-living organic carbon with a size greater than the limit for soil organic matter (suggested 2 mm) and less than the minimum diameter chosen for dead wood (e.g. 10 cm), in various states of decomposition above or within the mineral or organic soil. Live fine roots above the mineral or organic soil (of less than the minimum diameter limit chosen for below-ground biomass) are included in litter where they cannot be distinguished.
**Belowground carbon**	All carbon contained in live roots. Fine roots of less than (suggested) 2 mm diameter are often excluded because these often cannot be distinguished empirically from soil organic matter or litter.
**Soil carbon**	Includes organic carbon in mineral soils to a specified depth chosen by the country. Live and dead fine roots and dead organic matter within the soil, that are less than the minimum diameter limit specified (suggested 2 mm), are included with soil organic matter where they cannot be distinguished.

Land cover specific tier definitions are also available.

Currently, sampling effort is largely focussed on aboveground live carbon pools [15], [17]. However, the importance of the remaining IPCC carbon pools (litter, coarse woody debris, belowground, and soil carbon – see Table 1) is being increasingly recognised [18]–[21]. The size of these carbon pools is often estimated from ratios relating each pool to aboveground carbon stock [12], [15], [22]. Effort should also be made to capture an estimate of the uncertainty in values, although many studies omit this crucial step [11], [23].

**Figure 1:**
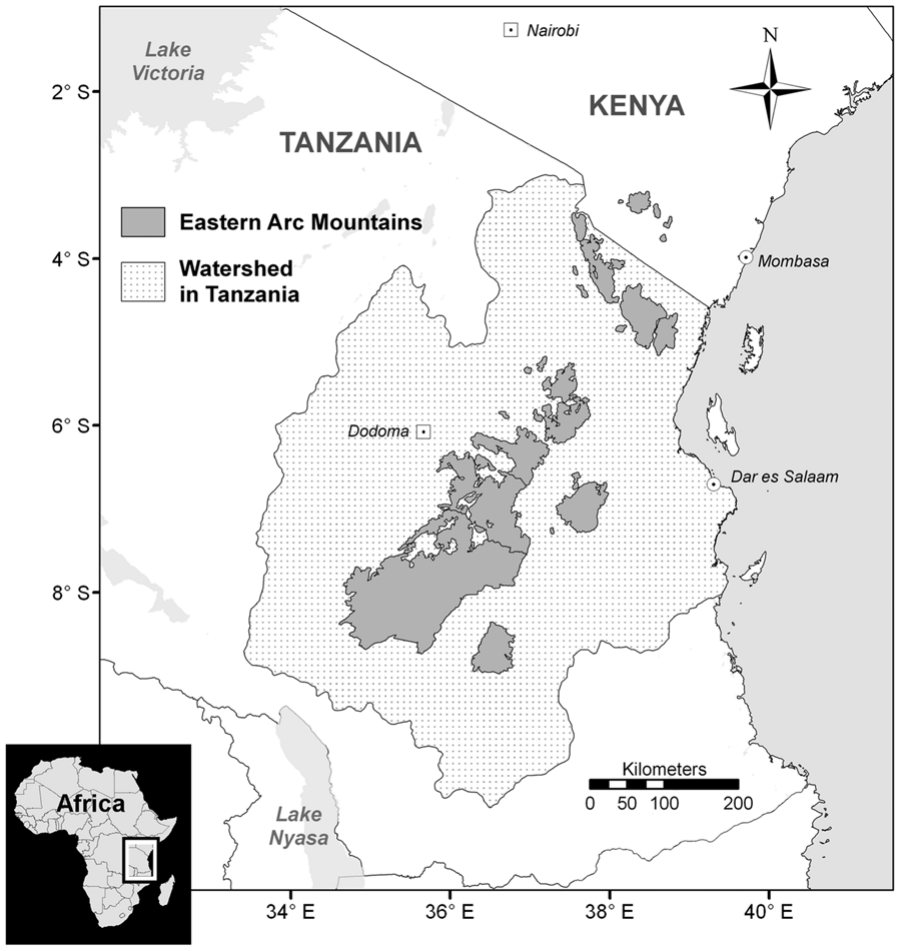
The Eastern Arc Mountains of Tanzania and Kenya [**30**]. The study area is the Eastern Arc watershed in Tanzania [29].

We use the watershed of the Eastern Arc Mountains in Tanzania (EAM), spanning 33.9 million ha (Figure 1), to derive regional carbon storage estimates using our method. At present, six previously published estimates, using a variety of methods, give a wide range of carbon storage estimates for our study area (Table 2). The lowest value given is derived from MODIS (1 km^2^ resolution) and LiDAR data plus limited ground observations, used to estimate the distribution of aboveground live carbon stored in Africa in 2000, giving a Tier 1 estimate of 0.34 Pg C for our study area. This estimate is for aboveground live carbon only, omitting the other four IPCC carbon pools, and utilises continental, not country, specific data and allometric equations. Following a critique of these methods [24], a recent revision has been published that fully accounts for disturbance, using inventory data, MODIS imagery and GLAS LiDAR data at a 500 m resolution to, surprisingly, provide the highest estimate of 2.03 Pg C for aboveground live carbon within our study area [25].

**Table 2:** Carbon stored within the study area for the year 2000 as estimated by this and previous studies (95% CI given in brackets).

Study	Aboveground live carbon storage, Pg (95% CI range)	Litter carbon storage, Pg (95% CI range)	Coarse woody debris carbon storage, Pg (95% CI range)	Belowground live carbon storage, Pg (95% CI range)	Aboveground live and belowground live carbon storage, Pg (95% CI range)	Soil carbon storage, Pg (95% CI range)	Total carbon storage, Pg (95% CI range)
Original	1.58 (1.56–1.60)	0.15 (0.14–0.15)	0.25 (0.24–0.25)	0.60 (0.59–0.61)	2.18 (2.15–2.21)	3.74 (3.43–4.05)	6.33 (5.92–6.74)
Harmonised	1.64 (1.52–1.76)	0.16 (0.15–0.17)	0.28 (0.26–0.30)	0.51 (0.47–0.55)	2.15 (1.99–2.30)	3.80 (3.77–3.82)	6.38 (6.33–6.43)
Baccini *et al* (2012)	2.03	N/A	N/A	N/A	N/A	N/A	N/A
Hurtt et al. (2006) HYDE-SAGE	0.63	N/A	N/A	N/A	N/A	N/A	N/A
Hurtt et al. (2006) HYDE	0.41	N/A	N/A	N/A	N/A	N/A	N/A
Baccini et al. (2008)	0.34	N/A	N/A	N/A	N/A	N/A	N/A
Ruesch & Gibbs (2008)	N/A	N/A	N/A	N/A	1.61	N/A	N/A
Saatchi et al. (2011)	0.83	N/A	N/A	0.26	1.09	N/A	N/A

Two carbon model outputs (HYDE and HYDE-SAGE) were presented by Hurtt et al. (2006) [26]. The HYDE-SAGE model, which uses more resolute cropland data than HYDE, produces an estimate of 0.63 Pg C for the study area (0.41 Pg C for the HYDE model) [26]. Through the use of the Miami LU ecosystem model, these estimates account for disturbance. These dynamic models could be used to provide Tier 3 estimates, however, the models do not utilise data or equations specific to our study area, instead using global (Tier 1) values to provide carbon estimates. Additionally, these models only provide estimates of aboveground live carbon storage.

The global vegetation map from the Global Land Cover 2000 Project (GLC2000; 100 ha resolution derived from SPOTVEGETATION satellite imagery [27]) is used in combination with carbon values produced by the IPCC to estimate Tier 1 carbon stock [11]. This approach accounted for disturbance only where vegetation categories were identified as disturbed (for example, burnt forests or cropland mosaics), but does present results for aboveground live and belowground carbon pools combined, estimating that 1.61 Pg C is stored within our study area [11]. Coarse woody debris, litter and soil pools are omitted. Saatchi et al (2011), using MODIS, SRTM and QSCAT to extrapolate inventory plot and GLAS LiDAR data, produces an estimate of 0.83 Pg C (Table 2) [28]. They provide estimates for both aboveground live and belowground carbon pools, omitting coarse woody debris, litter and soil, but accounting for disturbance. Estimates provided utilise continental data and allometric equations and so result in Tier 1 estimates. Both the GLC2000 based values and the Saatchi values are in the middle range of the six estimates [11], [28].

Considering all the studies together, none give estimates for all five IPCC carbon pools, and while some utilise local remotely-sensed data, they mostly do not include local data from on-the-ground. The result is estimates for aboveground live carbon storage across the EAM ranging from 0.34 Pg C to 2.03 Pg C (Table 2).

In this paper, we present a method of obtaining improved regional (Tier 2) estimates of carbon storage for all five IPCC carbon pools in data-sparse regions. Using a case study in eastern Tanzania we apply the resultant median values and 95% confidence intervals (CI) to a recent land cover map to calculate carbon stock for the year 2000. These figures are then compared to published estimates of carbon storage produced for the same study area in the same year. Our results suggest that by adopting the method presented here, countries currently using Tier 1 values may be able to generate Tier 2 values which can be easily updated and improved, incorporating inventory data as and when available, until data are sufficient to progress to a Tier 3 method.

## Methods

### Study Area

Our study area is the watershed of the EAM in Tanzania, covering 33.9 million hectares (Figure 1; see [29] for further details). The EAM themselves (5.2 million ha, as delimited in Platts et al., 2011 [30]) are nested within the broader study area and are considered a global priority for biodiversity conservation [31], with high levels of plant and animal endemism (including at least 96 vertebrate species and 471 vascular plant species) [32]–[34]. The watershed is a heterogeneous mix of cropland, savanna, miombo woodland and forest, and contains the administrative and commercial capitals of Dodoma and Dar es Salaam, respectively. The region provides numerous ecosystem services including carbon storage, water provision and regulation, maintenance of soil quality, reduction of erosion, regulation of run-off, stabilisation of local climate, conservation of cultural values (including traditional medicine), hydroelectricity generation and nutrient cycling [35]–[38]. As a United Nations REDD+ pilot country [7], a better understanding of the current carbon stock and distribution in Tanzania will likely inform policy choices.

### Overview

**Figure 2:**
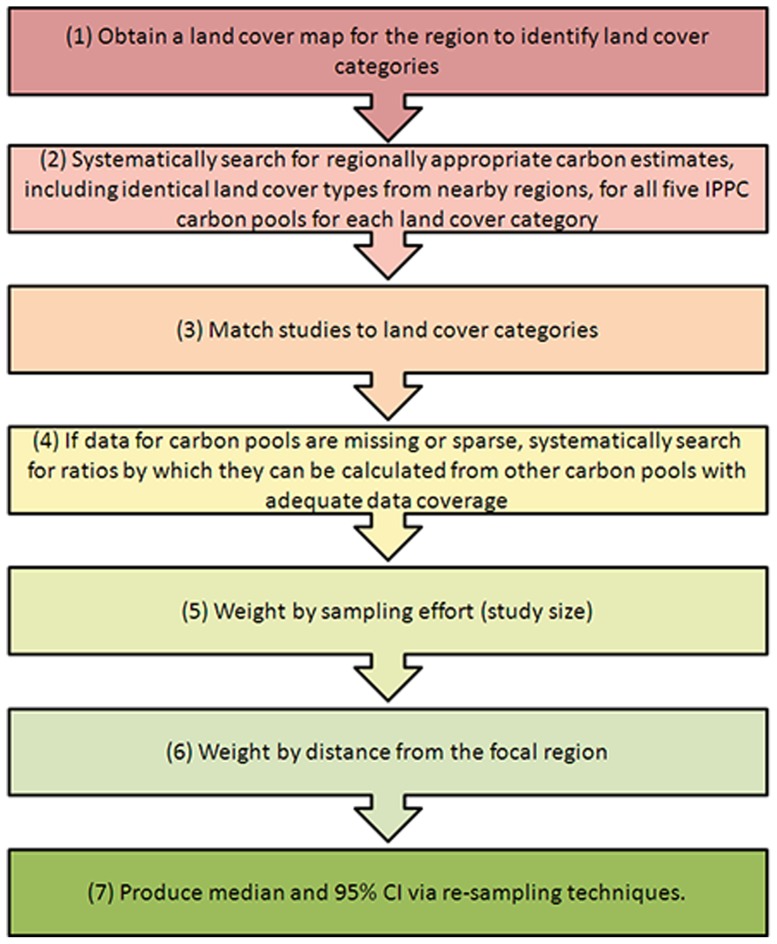
A summary of the seven stage method utilised here to produce regionally appropriate carbon estimates and 95% CI.

The method follows seven stages (Figure 2), summarised here and described in detail below: (1) Obtain a land cover map for the region to identify land cover categories; (2) Systematically search for regionally appropriate carbon estimates, including identical land cover types from nearby regions, for all five IPCC carbon pools for each land cover category; (3) Match studies to land cover categories; (4) If data for carbon pools are missing or sparse, then systematically search for ratios by which they can be calculated from other carbon pools with adequate data coverage; (5) Weight by sampling effort (study size); (6) Weight by distance from the focal region; (7) Produce median and 95% confidence intervals (CI) using re-sampling techniques.

### (1) Land Cover Map

**Figure 3:**
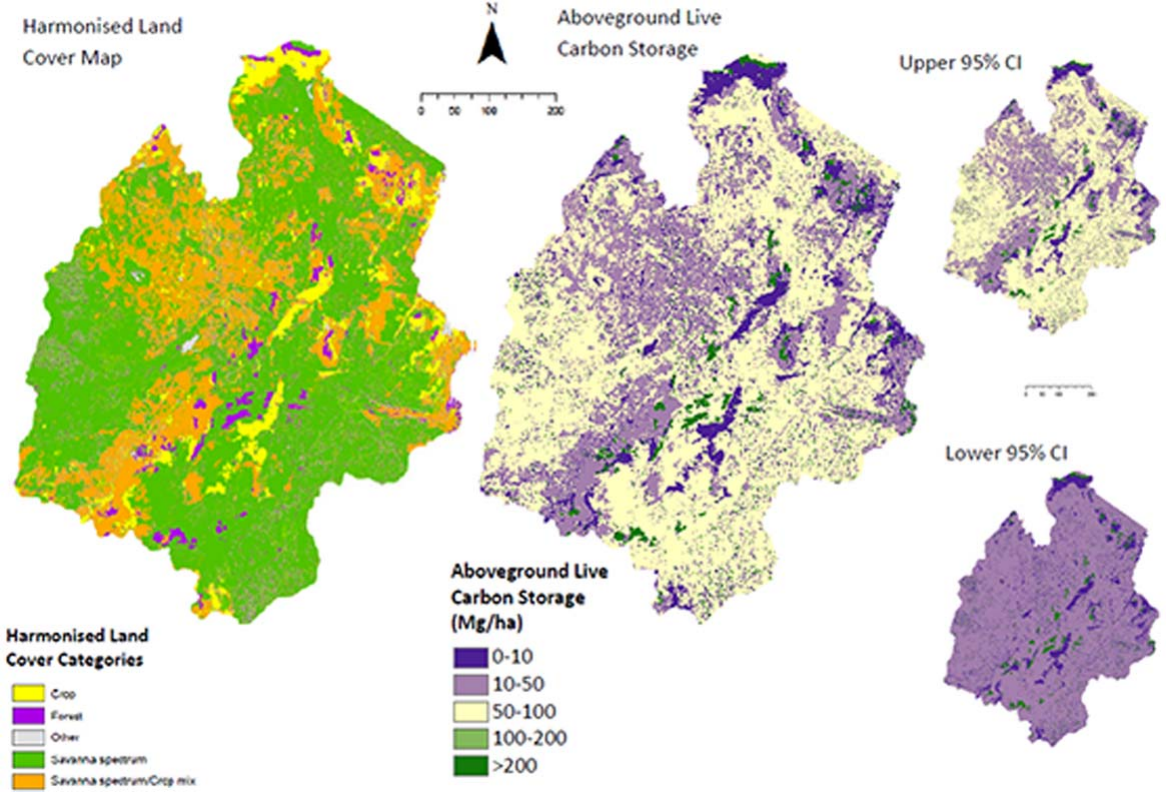
The spatial distribution of aboveground live carbon storage and associated pixel errors within the study area, based on combining the harmonised land cover map with our regionally appropriate carbon values (**Table 3**). Maps derived from the original land cover categories are shown in Figure S1.

We obtained a land cover map of 1 ha resolution, derived from a 1997 survey of LANDSAT and SPOT images undertaken for the Tanzanian government [39], with validation by local experts to ensure the map was applicable for the year 2000 [29]. This map recognised 30 land cover classes, termed hereafter ‘original land cover categories’. Since globally available land cover products (e.g. GlobCover, MODIS etc) typically describe fewer and/or different land cover categories, we investigated the effect that an alternative categorisation would have on the resulting carbon estimates. We therefore reclassified regional land cover according to four major categories that all land-cover schemes conform to, termed hereafter ‘harmonised land cover categories’. These are: forest (high carbon density tree-dominated systems, including montane forest, coastal forest, mangroves and tree plantations), savanna spectrum (medium carbon density mixed tree and grass systems, including miombo woodland, savanna, bushland and grassland), crop (anthropogenic arable systems) and other (largely dominated by low carbon systems, such as semi-desert and snow) (Figure 3, Figure S1, Table S1). Any mixed crop system category (grassland with scattered cropland or bushland and woodland equivalents) were split equally between crop and savanna-spectrum categories.

### (2) Carbon Data Search

Data from the literature were obtained by systematically entering search terms into Google Scholar, JSTOR and ISI Web of Knowledge search engines. The search terms combined both the 34 (original and harmonised) land cover category and carbon pool names (above ground, coarse woody debris, litter, root, belowground, soil, biomass, carbon, yield) plus geographical terms (Eastern Arc Mountains [EAM], Tanzania, East Africa, Africa). The bibliographies of all the sources we used for carbon data were checked for additional relevant references and data. To be included, carbon storage or biomass estimates must be reported, with studies excluded if the land use type was absent from our study site (e.g. temperate grasslands). For some agricultural land covers, yield data were more widely available and these were converted to standing crop biomass using published equations [40]–[42], the exception being sugarcane, where almost the entire crop is utilised (so annual yield was assumed to be equal to the aboveground live biomass). In total, 45 published papers fulfilled the search criteria (Table 3, Table S2, Table S3, References S1).

**Table 3:** Tier 2 carbon values for all five IPCC carbon pools using the harmonised land cover categories.

Description	Area (M ha)	Aboveground live (Mg ha^−1^)	Litter (Mg ha^−1^)	Coarse woody debris (Mg ha^−1^)	Belowground live (Mg ha^−1^)	Soil (Mg ha^−1^)	TOTAL (Mg ha^−1^)	References
**Forest**	0.96	221.9 (209.1–236.5; 8.7%; n = 1703)	10.9 (10.3–11.6; 8.6%)	13.1 (12.3–13.9; 8.7%)	54.2 (51.1–57.8; 8.7%)	116.8 (113.7–119.9; 3.7%)	416.9 (396.5–439.6; 7.3%)	[15], [48], [52], [66]–[81] & unpublished data
**Savanna spectrum**	26.02	28.6 (19.8–43.9; 61.5%; n = 185)	3.0 (2.0–4.7; 65.5%)	5.1 (3.5–7.9; 62.5%)	9.1 (6.4–13.8; 59.4%)	116.2 (112.6–120.2; 4.6%)	162.1 (144.4–190.5; 20.6%)	[48], [53], [58], [59], [68], [72], [82]–[94] & unpublished data
**Crop**	6.69	3.3 (1.9–5.8; 86.3%; n = 14)	0.1 (0.1–0.2; 83.0%)	0.3 (0.2–0.5; 85.8%)	0.9 (0.5–1.6; 86.2%)	123.3 (118.8–128.1; 5.3%)	127.9 (121.5–136.1; 8.2%)	[48], [59], [95]–[98]
**Other**	0.19	2.0 (2.0–4.9; 148.9%; n = 6)	0.6 (0.6–1.6; 151.5%)	0.8 (0.8–1.9; 148.9%)	0.0 (0.0–0.0; 0.0%)	97.2 (92.5–102.3; 7.1%)	100.6 (95.9–110.7; 11.0%)	[48], [59], [85] & unpublished data

Confidence limits, percent error and sample size (n) are shown in brackets. Confidence intervals were calculated via sampling with replacement (see text for details). Original land cover categories estimates are shown in Table S3.

These published data were supplemented with unpublished data. Local and international agencies working in the EAM region were contacted and written memoranda of understanding were agreed (outlining the investigations to be undertaken and the data sharing procedure), enabling a total of 2,462 tree inventory plots to be sourced. Aboveground live tree biomass (≥10 cm diameter) was estimated using an allometric equation for woodland (‘dry forest’) and forest (‘moist forest’) which uses estimates of diameter, wood density (from a global database [43] matched to stems using standard taxon-based procedures [44]) and tree height (using a height∶diameter relationship from African forests [15]) to determine dry mass [45]. The carbon content of vegetation varies relatively little across a wide variety of plant and tissue types [46], [47]. As such, carbon was assumed to be 50% of dry biomass, consistent with other studies conducted in Africa [15]. Additionally, it was assumed that the carbon values reported in published and unpublished studies were representative of the appropriate land cover category regardless of the date of measurement within the year.

### (3) Coupling Land Cover Categories and Carbon Values

Each data point was assigned to the appropriate land cover category by matching the site description in the carbon data with the land cover categories present in this study (Table 3, Table S3). After this process, it was evident that most studies (91.8%) considered aboveground live carbon storage only. This resulted in 63.3%, 36.7% and 30.0% of land cover categories containing more than five data points for aboveground live, belowground and soil carbon pools respectively.

### (4) Supplementing Data

Hence, despite a wealth of aboveground live inventory data for forest land cover categories, there are very few data for many land cover types in our study area (Table 3, Table S3). Furthermore, when conducting biomass inventories, it is not possible to sample every portion of aboveground live carbon. Of the studies reporting aboveground live carbon storage, most (90.8%) reported only the *measured* aboveground live carbon storage (for example, the carbon stored in trees with a diameter over 10 cm). In order to obtain the aboveground live carbon value for these studies, it is necessary to estimate the unmeasured aboveground live component. Thus, we undertook a second systematic literature search (in the same manner) to locate the ratios between aboveground live carbon storage and the other pools (including unmeasured aboveground live carbon but excluding soil carbon, which does not scale with aboveground carbon). Measured and unmeasured aboveground carbon pools were combined additively to give the traditional IPCC aboveground live carbon pool.

We obtained soil carbon values from the Southern Africa SOTER database [48], [49]. SOTER was chosen because it is freely available and contains regionally obtained data to a standard depth of 1 m. Values from the literature were also available [50]–[52], but the varying depths of each study made comparisons difficult. SOTER data were extracted by spatially matching the soil characteristics with the original and harmonised land cover categories of our land cover map. This procedure was followed for all vegetation types except for permanent swamp, because the SOTER database did not contain any appropriate regional cores and so a locally derived value of 683 Mg ha^−1^ was used [53].

### (5) Sampling Effort Weighting

In order to combine the carbon estimates from individual studies into a single value for each land cover category, each carbon value was weighted by the square root of the sum of number of hectares surveyed, ensuring that larger, studies contribute more to a final best estimate carbon value. Studies were weighted by sampling effort because confidence in biomass estimation increases with the number of hectares surveyed [54], [55]. If information on the study area was unavailable then we assumed the study had the same sample size as the median of those studies from the same land cover type. When fewer than five studies with sample sizes were available, the study size was assumed to be one hectare (this assumption was required for the mangrove, savanna, wetland and ‘other vegetation’ types).

### (6) Regional Weighting

Mean carbon storage for each land cover class was further weighted by the distances of individual carbon estimates from our study area. We first defined a hierarchy of four non-overlapping regions: our study area, outside our study area but within East Africa, elsewhere in Africa, and elsewhere in the world. Second, we took a square root weighting approach to the four regions. we took the square root of the weighting given to an area at the higher level in the hierarchy of regions, i.e. a carbon storage value from East Africa but from outside our study region was given the square root of the weighting of a study inside our study region. Then carbon storage value from outside East Africa, but inside Africa was given the square root of the weighting given to a value from inside East Africa, but outside our study region. Finally, a study from outside Africa was assigned the square root of the weighting of a study from Africa, but outside East Africa. The weightings are therefore 256∶16∶4∶2 for plots within the four areas. Thus plots within our study area were weighted much higher than those studies from further afield, while not ignoring data from outside the region, which is helpful as some land cover classes have little or no regional data. For aboveground live carbon storage values, 24 of the 34 land cover categories had less than five sample values specific to our study area. This reduced to 16, 13 and 11 land cover types respectively as data from the other regions were added. Hence, using all data in this way allowed carbon values and 95% CI to be obtained for all land cover types. These regional and previously described study size weightings were combined multiplicatively.

### (7) Derive Carbon Estimates

Derivation of carbon estimates occurred in two stages: (1) the production of carbon estimates and associated confidence intervals for each land cover type, and (2) the application of these values to our land cover map to produce landscape scale estimates of carbon storage. Firstly, the carbon value inputs into each land cover were sampled with replacement 10,000 times to produce the median weighted carbon value and 95% confidence limits (using R 2.12.1 [56]). These were mapped at a one hectare resolution in ArcGIS v9.3.1 [57] (Figure 3, Figure S1). Secondly, estimates of total landscape carbon storage were made by allocating each pixel in the map a randomly selected value within the appropriate pixel 95% CI. This process was performed 10,000 times and the median landscape carbon storage value and 95% CI were obtained.

## Results

Estimate carbon values from our methodology are given in Table 3 and Table S3. Using our approach, sub-montane forest is calculated to contain the most aboveground live carbon per unit area (283 [252–329] Mg ha^−1^), followed by montane forest (228 [190–286] Mg ha^−1^), lowland forest (207 [195–220] Mg ha^−1^), upper montane forest (202 [73–332] Mg ha^−1^) and forest mosaic (187 [174–201] Mg ha^−1^) (Table S3). This pattern was consistent when all carbon pools were combined, except that permanent swamp became the most carbon-dense land cover due to its large pool of soil carbon.

For forest, the aboveground live carbon pool was the largest, representing 53% of the total carbon stored in this ecosystem. Soil and belowground carbon pools were also substantial in forest ecosystems, containing 28% and 13% of total carbon stored respectively (Table 3). In savanna ecosystems, the soil carbon pool was most substantial, representing 72% of the total carbon stored. Crop and ‘other vegetation’ ecosystems store over 96% of their total carbon within the soil (Table 3).

For the 30 original land cover categories, the aboveground live carbon pool had a mean percentage error of 44±15%. However, when harmonised categories were used, this rose to 63±9% as a result of the smaller number of broader categories. Some land cover categories have high levels of uncertainty for total carbon values (most notably mangroves [±103%], sugar cane [±70%] and upper montane forest [±68%]), and some showed lower uncertainty (permanent swamp [±7%], bushland with scattered cropland [±9%] and lowland forest [±10%]) (Table 3, Figure S2, Table S3).

Assigning the carbon values to the land cover map indicates that 1.58 (1.56–1.60) Pg C was stored in the above ground live vegetation in the year 2000 in the study region using the original land cover categories (Figure 3; Table 2) and 1.64 (1.52–1.76) Pg C for the harmonised land cover categories. Woodland and bushland contributed most to the total stored aboveground live carbon in the study region. Specifically, open woodland stored the most aboveground live carbon (0.54 [0.45–0.65] Pg C over 9.6 million ha); followed by bushland (0.32 [0.16–0.55] Pg C over 5.0 million ha) and closed woodland (0.23 [0.15–0.28] Pg C over 1.8 million ha). However, when all carbon pools are considered the total carbon storage across the Eastern Arc drainage basin is 6.33 (5.92–6.74) Pg C using original land cover categories and 6.38 (6.33–6.43) Pg C for the harmonised land cover categories (Table 2). Considering the 30 original land cover classes, and all five carbon pools combined, the land cover were still dominated by open woodland (1.89 [1.67–2.12] Pg C) and bushland (1.07 [0.75–1.52] Pg C); now followed by grassland (0.79 [0.54–0.84] Pg C over 5.2 million ha).

## Discussion

Climate change mitigation schemes such as REDD+ need reliable, low-cost and repeatable estimates of carbon storage, ideally based on existing data. Our results suggest that the easiest and most commonly used approach of using global carbon storage values (Tier 1) can potentially result in large errors (generally, underestimation of carbon stocks by 26–78% in our study area). This poor performance is aggravated by the fact that uncertainty is seldom quantified for such values. The method we presented is cost and time efficient, while compliant with Tier 2 standards. Using it we estimate theaboveground live carbon storage for the study area in the year 2000 is 1.58 (1.56–1.60) Pg C for the original land cover categories, considerably greater than most previous estimates which have a mean of 0.85 Pg C (Table 2) [23], [26]. Our study is in close agreement with the previous result of Ruesch and Gibbs (2008) [11]. The recent Baccini et al (2012) carbon map is the only study to give a higher estimate than ours (Table 2) [25]. It is perhaps unsurprising that our estimates are close to those of Baccini et al. (2012), given that Tanzania was one of the multiple locations used to develop their regression models [25].

Here, we focussed on producing regionally appropriate carbon values for land cover types within our study area, whilst the studies we have compared our results to have attempted to map carbon over much larger scales. Thus, our estimates are regionally appropriate and error-bounded, fulfilling Tier 2 approach criteria (Table 1). Hence, the possible underestimation of some previous estimates in comparison to this study may indicate that eastern Tanzania has higher carbon storage than generally thought. However, when carbon values for land cover categories in this study are compared to similar land cover types elsewhere, the values appear to be in broad agreement (Table 3, Table S3) [11], [58], [59]. The carbon values used by both Hurtt et al. (2006) and Baccini et al. (2008) are substantially lower for comparable land cover categories than those in this study and Ruesch and Gibbs (2008) [11], [23], [26], suggesting that the two former approximations of carbon storage may be systematically underestimated [24]. Given the policy relevance of the carbon content of tropical vegetation, notably via REDD+, the possibility of such methodological errors should be an area of urgent further investigation. Further differences arise due to the higher resolution of this study (allowing for the identification of smaller fragments of forest, for example) which may have led to the substantial differences in the estimates of carbon storage within the highly heterogeneous landscape of our study area (Table 2). It should be noted that, whilst our study contains data from both pristine and disturbed habitats, there is a bias towards undisturbed habitats. Although the landscape is known to include significant habitat degradation, preliminary investigations to produce a ‘Tier 3’ regression model (i.e. explicitly accounting for disturbance and climatic variation) the same data give landscape carbon storage estimates higher than most previous studies. For example, if the lower 95 CI limit for each land cover category was used, indicating that every location showed disturbance, we would estimate the study area contained 1.06 Pg and 1.20 Pg of aboveground live carbon, using original and harmonised land cover categories respectively. These values are still substantially greater than those from most previous studies (Table 2). It is important that further work investigates the role of disturbance, edaphic and climatic variations as all three are known to affect carbon storage within our study area [30], [60]. This will be particularly important in estimating future carbon storage as east Africa is predicted to become both warmer and wetter, potentially increasing the landscape carbon storage [61]. However, this effect may be negated by the rising human population and associated demand on natural resources [62], which could lead to increased degradation and land cover change from high carbon systems to those with less carbon (for example, from savanna to agriculture [Table 3]).

Previous studies have only focussed on aboveground live and belowground live carbon pools [11], [23], [26], [28] and by selecting the relevant carbon pools we were able to make direct comparisons. Our study is unique in providing estimates for all five IPCC carbon pools for eastern Tanzania. Our results show that soil carbon makes up almost 60% of the total carbon stored, over double that represented by aboveground live carbon, and so emphasise the importance of investigating all five IPCC carbon pools.

Typically, land cover types of lower carbon density are less well studied. For instance, research within Tanzania has typically focussed on forests, which hold the most aboveground live biomass per unit area but, when all carbon pools are considered, permanent swamp - a poorly known land cover type - holds the most carbon per hectare. Furthermore, within our study region, other land cover categories span a greater area than forest. The systems storing the greatest amount of carbon, within our study region, are neither those land cover types that have the largest carbon store per unit area, nor the most extensive, but are those that are reasonably extensive with relatively high carbon storage per unit area. This result indicates that, on a landscape scale, carbon stored in woodland is extremely important. This ecosystem is currently highly utilised by the local population, resulting in rapid degradation [63], [64].

Overall, while there is high uncertainty in 1 ha pixel-size estimates, there are narrow confidence intervals around our landscape estimates. This is typical of studies where estimates of error are provided (see Saatchi et al. 2011 for an example [28]) and is a result of both the large study area and the small pixel size. When averaged across a large number of pixels, pixel error is mostly negated as underestimates in one part of the landscape are counterbalanced by overestimates in other parts. These estimates, however, may give a false sense of confidence if sources of error were directional, for example if sampling was biased towards undisturbed habitats. Thus, our weighting system has potential to introduce some bias, particularly the regional weightings which are somewhat arbitrary as (1) our four regions are not unambiguously clearly defined units, and (2) our square-root of approximate distance weightings are a first-order estimate. However, both on a pixel and a landscape level, unweighted results do not alter our overall conclusions (Table S4).

Several land cover categories show a disproportionally high level of error, indicative of both high natural carbon storage heterogeneity and low levels of sampling (Table 3, Table S3). Indeed, some land cover types within our study are relatively data-poor. However, the dominant land cover types within our study site are better sampled and show smaller errors, thus our conclusions are likely robust to both natural heterogeneity and data scarcity in some land cover types (Table 3, Table S3, Figure S2). The high natural variation observed in some well-sampled land cover categories illustrate that look-up table methods (Tiers 1 and 2) are oversimplified and hence disturbance and climate effects on carbon storage should be taken into account where data allow [16]. Litter, coarse woody debris, and below ground carbon pools all show similar levels of error to above ground live carbon because they are all derived from the latter pool using published ratios. Within this study, soil carbon appears to have a low uncertainty, despite being known to be extremely heterogeneous [13], [65], because of limited data availability. . Only 54 soil cores were used to produce the SOTER map for Tanzania [48], [49], an average of less than two per land cover category. Hence, much like litter, coarse woody debris, and below ground carbon, soil carbon in Tanzania (as elsewhere) requires much further research to improve future carbon estimates.

## Conclusions

We have presented a method of producing error-bounded, carbon values that conforms to IPCC Tier 2 reporting requirements. By coupling land cover classifications with systematic data searches it is possible to produce more regionally appropriate values despite the conditions of sparse local data that exist for most of the tropics. This method yields estimates for all five IPCC carbon pools, at low cost, and in manner which can be continually updated and improved, incorporating new studies and inventory data as and when they become available. Such regional carbon storage estimates have the potential to affect regional conservation and research priorities. Displaying uncertainties associated with these values transparently enables identification of critical areas of future research. Additionally, by more explicitly acknowledging natural variation and data scarcity, the method helps ensure that the uncertainties and limitations are conveyed to policy makers.

## Supporting Information

Figure S1 The spatial distribution of aboveground live carbon storage and associated pixel errors within the study area, based on combining the original land cover map with our regionally appropriate carbon values (Table 3).
(TIF)

Figure S2 The spatial distribution of the size of the cell 95% CI (expressed as a percentage) for the aboveground live carbon pool, using both original land cover categories.
(TIF)

Table S1 Original and harmonised land cover categories.
(DOCX)

Table S2 Ratios used in the derivation of understudied aboveground carbon pools.
(DOCX)

Table S3 The carbon values, confidence limits and percent error for all five IPCC carbon pools using the original land cover categories. M - Median carbon storage (Mg ha^−1^); lCI - Lower 95% confidence interval of carbon storage (Mg ha^−1^); uCI - Upper 95% confidence interval of carbon storage (Mg ha^−1^); % - Percent error (%); n – Sample size).
(DOCX)

Table S4 The carbon values and confidence limits for the aboveground live carbon pool using the original and harmonised land cover categories without any form of weighting. These values are not significantly different from the weighted values shown in Table 3 and Table S3 when a paired t-test is performed (p-value<0.693). The range of landscape values derived from these (1.22 [0.91–1.61] Pg C and 1.70 [1.46–1.98] Pg C for original and harmonised land cover categories respectively) overlap those derived from the weighted values and are also significantly higher than previous estimates (Table 2). (Area (million ha); M - Median carbon storage (Mg ha^−1^); lCI - Lower 95% confidence interval of carbon storage (Mg ha^−1^); uCI - Upper 95% confidence interval of carbon storage (Mg ha^−1^); n – Sample size).
(DOCX)

References S1 Citations for the data included in Tables S2, S3, S4.
(DOCX)
