# Supplementary figures and images for: Towards Regional, Error-Bounded Landscape Carbon Storage Estimates for Data-Deficient Areas of the World
Source: PLoS One. 2012 Sep 14;7(9):e44795. doi: 10.1371/journal.pone.0044795 (PMC3443093; doi:10.1371/journal.pone.0044795)

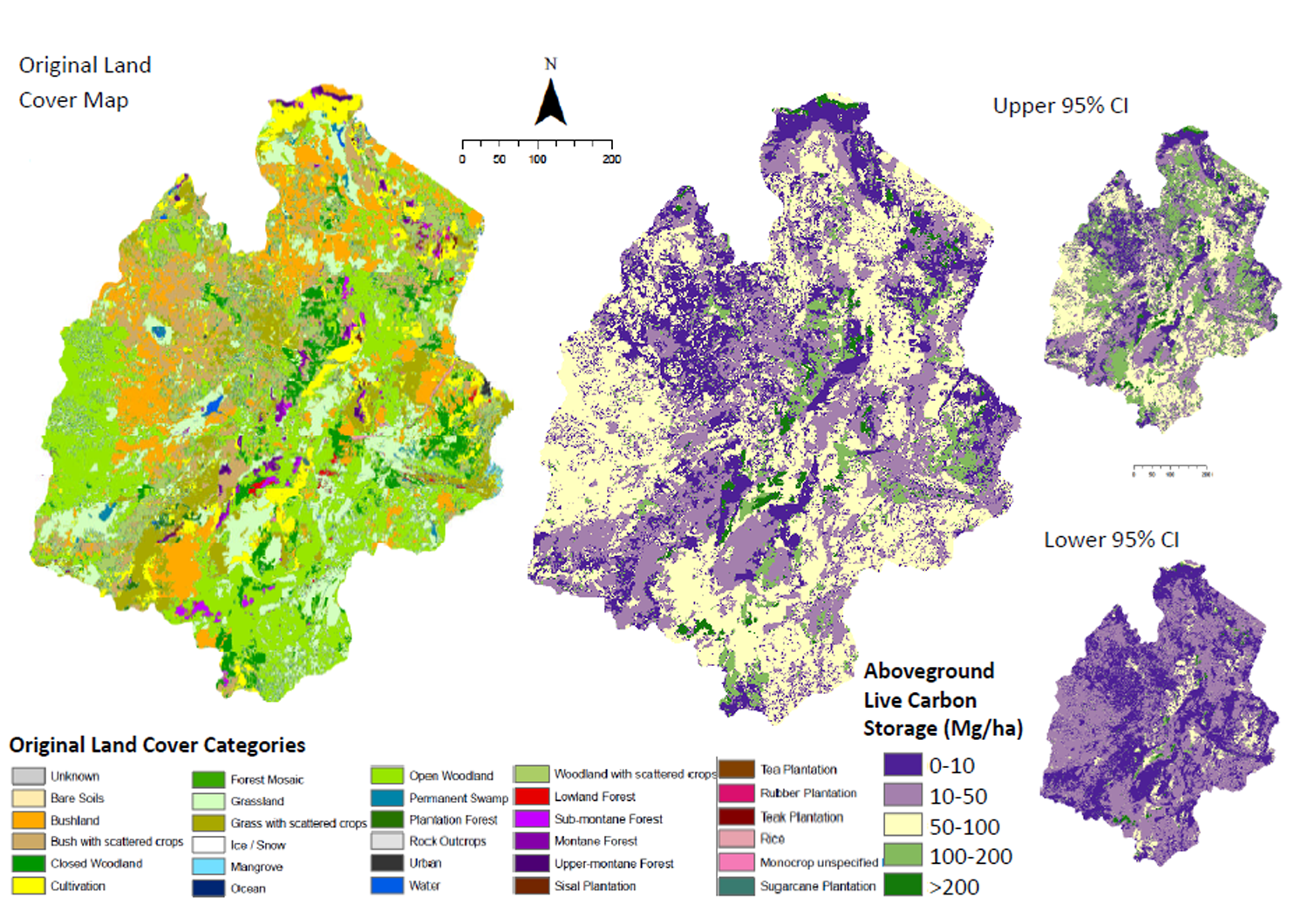

Supplement: Figure S1 — The spatial distribution of aboveground live carbon storage and associated pixel errors within the study area, based on combining the original land cover map with our regionally appropriate carbon values (Table 3). (TIF) [file pone.0044795.s001.tif]

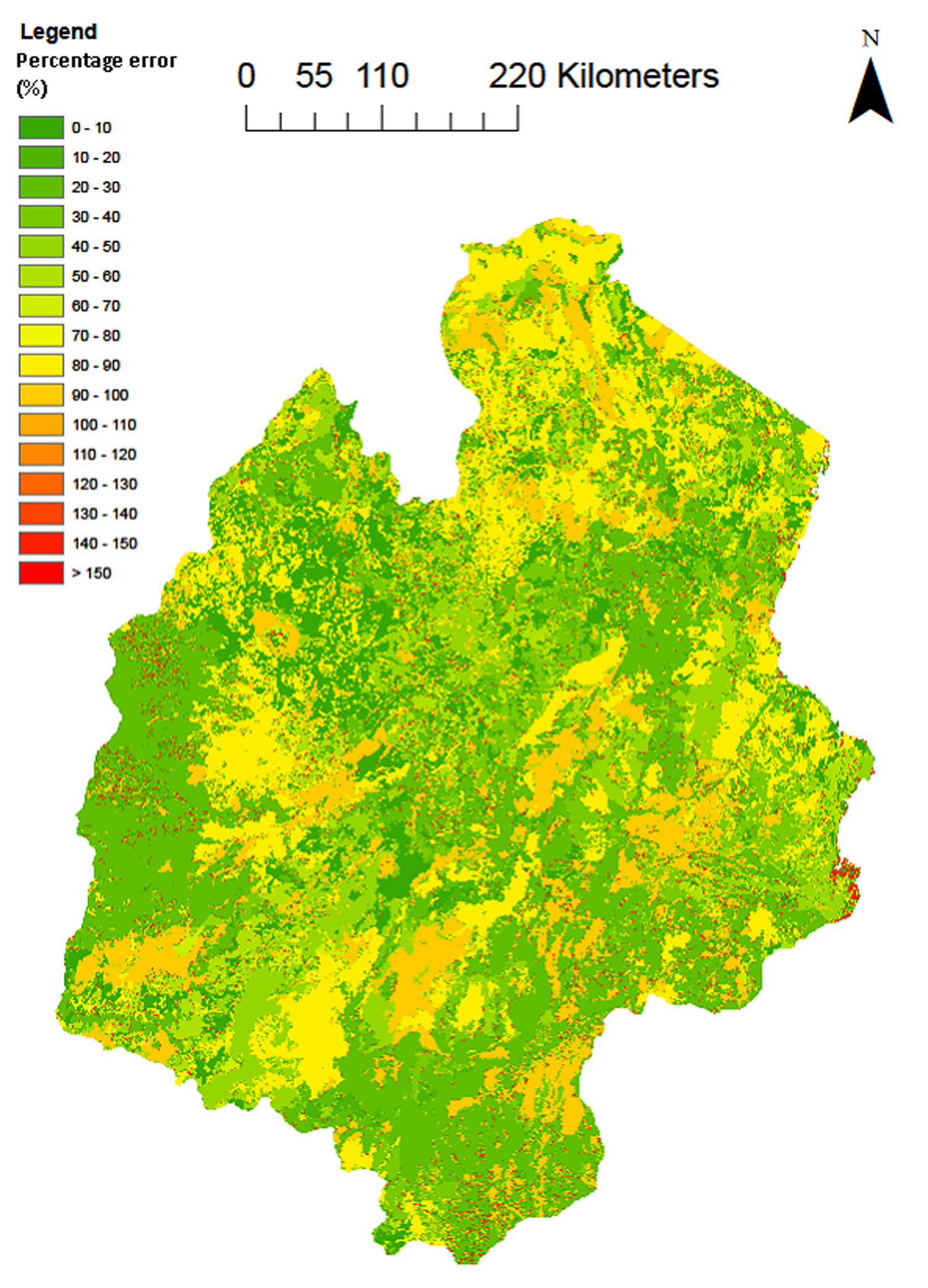

Supplement: Figure S2 — The spatial distribution of the size of the cell 95% CI (expressed as a percentage) for the aboveground live carbon pool, using both original land cover categories. (TIF) [file pone.0044795.s002.tif]
