# Supplementary material for: Towards Regional, Error-Bounded Landscape Carbon Storage Estimates for Data-Deficient Areas of the World
Source: PLoS One. 2012 Sep 14;7(9):e44795. doi: 10.1371/journal.pone.0044795 (PMC3443093; doi:10.1371/journal.pone.0044795)
Supplement: Table S1 — Original and harmonised land cover categories. (DOCX) [file pone.0044795.s003.docx]

**Table S1** – Original and harmonised land cover categories.

| **Harmonised land use category** | **Original land use category** | |
| --- | --- | --- |
| Forest | - 22 Montane Forest - 21 Sub-montane forest - 20 Lowland Forest - 23 Upper-montane forest | - 11 Mangrove forest - 15 Plantation Forest - 27 Teak plantation - 26 Rubber plantation |
| Savanna spectrum | - 5 Closed Woodland - 0.5*(19 Woodland with scattered cropland) - 3 Bushland - 0.5*(4 Bushland with scattered cropland) - 7 Forest mosaic | - 13 Open Woodland - 8 Grassland - 14 Permanent Swamp - 0.5*(9 Grassland with scattered cropland) |
| Crop | - 24 Sisal plantation - 25 Tea plantation - 6 Cultivation - 0.5*(19 Woodland with scattered cropland) - 0.5*(9 Grassland with scattered cropland) | - 0.5*(4 Bushland with scattered cropland) - 28 Rice plantation - 29 Monocrop unspecified - 3 Sugarcane plantation |
| Other | - 1 Unclassified - 2 Bare Soils - 10 Ice - 12 Ocean | - 16 Rock outcrops - 17 Urban Area - 18 Water |
