# Supplementary material for: Towards Regional, Error-Bounded Landscape Carbon Storage Estimates for Data-Deficient Areas of the World
Source: PLoS One. 2012 Sep 14;7(9):e44795. doi: 10.1371/journal.pone.0044795 (PMC3443093; doi:10.1371/journal.pone.0044795)
Supplement: Table S2 — Ratios used in the derivation of understudied aboveground carbon pools. (DOCX) [file pone.0044795.s004.docx]

**Table S2 –** Ratios used in the derivation of understudied aboveground carbon pools

| **Vegetation Group** | **Unmeasured vegetation types (%)** | **References** | **Vegetation biomass below the DBH threshold** ^1^ | **References** | **Litter** | **References** | **Coarse woody debris** | **References** | **Belowground** | **References** |
| --- | --- | --- | --- | --- | --- | --- | --- | --- | --- | --- |
| **Bush** | 0.0011 | [[1](#_ENREF_1)] | 0.0021*DBH | [[2](#_ENREF_2),[3](#_ENREF_3)] | 0.1546 | [[1](#_ENREF_1),[4](#_ENREF_4),[5](#_ENREF_5)] | 0.2400 | [[1](#_ENREF_1),[4](#_ENREF_4),[6](#_ENREF_6),[7](#_ENREF_7)] | 0.3177 | [[1](#_ENREF_1),[8](#_ENREF_8)] |
| **Crop** | 0.1987 | [[9](#_ENREF_9),[10](#_ENREF_10),[11](#_ENREF_11)] | N/A | N/A | 0.1987*0.2039 | N/A ^2^ | 0.1987*0.4769 | N/A ^3^ | 0.3209 | [[12](#_ENREF_12),[13](#_ENREF_13),[14](#_ENREF_14)] |
| **Forest** | 0.0043 | [30-32] | 0.0052*DBH | [[15](#_ENREF_15)] | 0.0518 | [[16](#_ENREF_16),[17](#_ENREF_17)] | 0.0622 | [[6](#_ENREF_6),[7](#_ENREF_7),[16](#_ENREF_16),[17](#_ENREF_17),[18](#_ENREF_18)] | 0.2578 | [[1](#_ENREF_1),[17](#_ENREF_17),[19](#_ENREF_19),[20](#_ENREF_20),[21](#_ENREF_21),[22](#_ENREF_22)] |
| **Grass** | 0.1987*0.5 | N/A ^4^ | N/A | N/A | 0.1987*0.5*0.2039 | N/A ^2^ | 0.1987*0.5*0.4769 | N/A ^3^ | 1.6 | [[23](#_ENREF_23)] |
| **Mangrove** | 0.0227 | [[24](#_ENREF_24)] | 0.0059*DBH | [[25](#_ENREF_25)] from [[26](#_ENREF_26)] | 0.5054 | [[24](#_ENREF_24),[27](#_ENREF_27),[28](#_ENREF_28)] | 0.0835 | [[29](#_ENREF_29)] | 0.2691 | [[29](#_ENREF_29),[30](#_ENREF_30)] |
| **Other** | N/A | N/A | N/A | N/A | 0.3250 | [[31](#_ENREF_31)] | 0.4 | [[6](#_ENREF_6)] | 0 | N/A ^5^ |
| **Savanna** | 0.3842 | [[11](#_ENREF_11),[32](#_ENREF_32),[33](#_ENREF_33)] | 0.0021*DBH | [[2](#_ENREF_2),[3](#_ENREF_3)] | 0.2039 | [[4](#_ENREF_4),[11](#_ENREF_11),[33](#_ENREF_33),[34](#_ENREF_34),[35](#_ENREF_35)] | 0.4769 | [[4](#_ENREF_4),[6](#_ENREF_6),[11](#_ENREF_11),[32](#_ENREF_32),[36](#_ENREF_36)] | 0.7437 | [[23](#_ENREF_23),[37](#_ENREF_37)] |
| **Wetland** | 0.0526 | [[38](#_ENREF_38)] | N/A | N/A | N/A | N/A | 0.2542 | [[39](#_ENREF_39)] | 1.9456 | [[40](#_ENREF_40),[41](#_ENREF_41)] |
| **Wood** | 0.2048 | [[32](#_ENREF_32),[33](#_ENREF_33),[42](#_ENREF_42)] | 0.0021*DBH | [[2](#_ENREF_2),[3](#_ENREF_3)] | 0.1145 | [[4](#_ENREF_4),[33](#_ENREF_33),[34](#_ENREF_34),[35](#_ENREF_35)] | 0.1274 | [[4](#_ENREF_4),[6](#_ENREF_6),[7](#_ENREF_7),[32](#_ENREF_32),[35](#_ENREF_35),[43](#_ENREF_43)] | 0.3856 | [[23](#_ENREF_23),[34](#_ENREF_34),[44](#_ENREF_44)] |

^1^ To incorporate the smaller trees in these studies it was assumed that the number of trees below 10cm diameter at breast height was a linear relationship. If data on the DBH threshold was unavailable it was assumed individually based on each paper in an ad hoc basis.

^2^ The measured vegetation in cropland and grassland is assumed to produce minimal litter. Hence the unmeasured woody part is the source of the litter and the ratio of this component to litter is assumed to be the same as savanna.

^3^ The unmeasured vegetation of both cropland and grassland is assumed to be woody and produce all of the coarse woody debris in the classification. The ratio of this component to coarse woody debris is assumed to be the same as that from savanna.

^4^ The unmeasured vegetation type ratio for grassland is assumed to be 50% ratio for crops.

^5^ The belowground component for the category ‘other’ is assumed to be zero.
