# Supplementary material for: Towards Regional, Error-Bounded Landscape Carbon Storage Estimates for Data-Deficient Areas of the World
Source: PLoS One. 2012 Sep 14;7(9):e44795. doi: 10.1371/journal.pone.0044795 (PMC3443093; doi:10.1371/journal.pone.0044795)
Supplement: Table S3 — The carbon values, confidence limits and percent error for all five IPCC carbon pools using the original land cover categories. M - Median carbon storage (Mg ha−1); lCI - Lower 95% confidence interval of carbon storage (Mg ha−1); uCI - Upper 95% confidence interval of carbon storage (Mg ha−1); % - Percent error (%); n – Sample size). (DOCX) [file pone.0044795.s005.docx]

**Table S3 –** The carbon values, confidence limits and percent error for all five IPCC carbon pools using the original land cover categories. M - Median carbon storage (Mg ha^-1^); lCI - Lower 95% confidence interval of carbon storage (Mg ha^-1^); uCI - Upper 95% confidence interval of carbon storage (Mg ha^-1^); % - Percent error (%); n – Sample size)

| Description | Area (M ha) | Aboveground live | | | | | Litter | | | | Coarse woody debris | | | | Belowground live | | | | Soil | | | | TOTAL | | | | References |
| --- | --- | --- | --- | --- | --- | --- | --- | --- | --- | --- | --- | --- | --- | --- | --- | --- | --- | --- | --- | --- | --- | --- | --- | --- | --- | --- | --- |
|  |  | **M** | lCI | uCI | % | **n** | **M** | lCI | uCI | % | **M** | lCI | uCI | % | **M** | lCI | uCI | % | **M** | lCI | uCI | % | **M** | lCI | uCI | % |  |
| 1 Unclassified | 0.00 | **0.0** | 0.0 | 0.0 | 0.0 | 0 | **0.0** | 0.0 | 0.0 | 0.0 | **0.0** | 0.0 | 0.0 | 0.0 | **0.0** | 0.0 | 0.0 | 0.0 | **100.9** | 85.8 | 120.3 | 24.3 | **100.9** | 85.8 | 120.3 | 24.3 | [[45](#_ENREF_45)] |
| 2 Bare Soils | 0.02 | **2.0** | 2.0 | 2.0 | 0.0 | 1 | **0.6** | 0.6 | 0.6 | 0.0 | **0.8** | 0.8 | 0.8 | 0.0 | **0.0** | 0.0 | 0.0 | 0.0 | **96.0** | 87.5 | 106.7 | 14.2 | **99.4** | 90.8 | 110.1 | 13.8 | [[45](#_ENREF_45)] & unpublished data |
| 3 Bushland | 5.04 | **62.9** | 31.3 | 109.6 | 89.6 | 15 | **9.6** | 4.8 | 16.6 | 88.3 | **15.0** | 7.5 | 26.0 | 88.9 | **19.8** | 10.0 | 34.4 | 88.7 | **104.8** | 95.4 | 115.3 | 13.4 | **212.1** | 149.1 | 301.8 | 51.7 | [[1](#_ENREF_1),[8](#_ENREF_8),[45](#_ENREF_45)] & unpublished data |
| 4 Bushland with scattered cropland | 3.41 | **8.2** | 8.2 | 8.2 | 0.0 | 1 | **0.3** | 0.3 | 0.3 | 0.0 | **0.6** | 0.6 | 0.6 | 0.0 | **2.2** | 2.2 | 2.2 | 0.0 | **106.6** | 100.1 | 114.5 | 9.6 | **117.8** | 111.3 | 125.8 | 8.7 | [[14](#_ENREF_14),[45](#_ENREF_45)] |
| 5 Closed Woodland | 1.82 | **125.2** | 84.4 | 153.5 | 39.7 | 104 | **11.8** | 8.0 | 14.4 | 39.3 | **21.1** | 14.2 | 25.8 | 39.4 | **39.8** | 27.0 | 48.6 | 39.2 | **103.5** | 97.5 | 109.8 | 8.4 | **301.4** | 231.0 | 352.1 | 28.8 | [[32](#_ENREF_32),[34](#_ENREF_34),[45](#_ENREF_45)] & unpublished data |
| 6 Cultivation | 2.46 | **3.3** | 1.9 | 5.8 | 87.2 | 14 | **0.1** | 0.1 | 0.2 | 83.8 | **0.3** | 0.2 | 0.5 | 84.9 | **0.9** | 0.5 | 1.6 | 86.9 | **118.8** | 110.7 | 128.0 | 10.3 | **123.4** | 113.4 | 136.1 | 13.1 | [[14](#_ENREF_14),[45](#_ENREF_45),[46](#_ENREF_46),[47](#_ENREF_47),[48](#_ENREF_48),[49](#_ENREF_49)] |
| 7 Forest mosaic | 0.05 | **187.0** | 173.5 | 201.1 | 10.4 | 573 | **9.3** | 8.6 | 10.0 | 10.5 | **11.2** | 10.4 | 12.0 | 10.4 | **45.7** | 42.5 | 49.2 | 10.5 | **125.5** | 90.6 | 174.6 | 48.0 | **378.7** | 325.5 | 446.9 | 22.8 | [[45](#_ENREF_45)] & unpublished data |
| 8 Grassland | 5.18 | **17.9** | 1.5 | 18.0 | 91.4 | 7 | **0.3** | 0.0 | 0.3 | 91.5 | **0.8** | 0.1 | 0.8 | 91.4 | **26.0** | 2.2 | 26.2 | 91.4 | **108.4** | 100.8 | 117.4 | 10.8 | **153.4** | 104.7 | 162.7 | 32.3 | [[8](#_ENREF_8),[12](#_ENREF_12),[45](#_ENREF_45),[50](#_ENREF_50),[51](#_ENREF_51)] |
| 9 Grassland with scattered cropland | 2.73 | **12.6** | 7.2 | 13.2 | 43.1 | 3 | **0.4** | 0.2 | 0.4 | 43.1 | **1.0** | 0.6 | 1.0 | 43.1 | **3.4** | 1.9 | 3.5 | 43.1 | **111.7** | 103.0 | 120.9 | 11.3 | **129.0** | 112.9 | 139.1 | 14.7 | [[12](#_ENREF_12),[45](#_ENREF_45),[46](#_ENREF_46)] |
| 10 Ice | 0.00 | **0.0** | 0.0 | 0.0 | 0.0 | 0 | **0.0** | 0.0 | 0.0 | 0.0 | **0.0** | 0.0 | 0.0 | 0.0 | **0.0** | 0.0 | 0.0 | 0.0 | **165.8** | 129.1 | 202.5 | 31.3 | **165.8** | 129.1 | 202.5 | 31.3 | [[45](#_ENREF_45)] |
| 11 Mangrove forest | 0.06 | **87.5** | 52.8 | 206.0 | 141.0 | 6 | **34.2** | 9.5 | 91.7 | 182.9 | **6.4** | 3.6 | 15.9 | 153.9 | **21.6** | 13.1 | 50.1 | 137.5 | **79.8** | 71.5 | 89.6 | 16.1 | **229.6** | 150.5 | 453.4 | 103.4 | [[20](#_ENREF_20),[29](#_ENREF_29),[30](#_ENREF_30),[45](#_ENREF_45),[52](#_ENREF_52),[53](#_ENREF_53)] |
| 12 Ocean | 0.00 | **0.0** | 0.0 | 0.0 | 0.0 | 0 | **0.0** | 0.0 | 0.0 | 0.0 | **0.0** | 0.0 | 0.0 | 0.0 | **0.0** | 0.0 | 0.0 | 0.0 | **98.1** | 84.5 | 113.7 | 21.1 | **98.1** | 84.5 | 113.7 | 21.1 | [[45](#_ENREF_45)] |
| 13 Open Woodland | 9.62 | **56.5** | 46.5 | 67.6 | 26.3 | 114 | **5.6** | 4.8 | 6.5 | 22.7 | **10.4** | 8.7 | 12.1 | 23.4 | **18.9** | 15.9 | 22.1 | 23.5 | **104.7** | 98.2 | 111.5 | 9.0 | **196.1** | 174.0 | 219.9 | 16.5 | [[32](#_ENREF_32),[34](#_ENREF_34),[37](#_ENREF_37),[44](#_ENREF_44),[45](#_ENREF_45),[48](#_ENREF_48),[54](#_ENREF_54),[55](#_ENREF_55)] & unpublished data |
| 14 Permanent Swamp | 0.18 | **23.5** | 11.9 | 35.0 | 69.5 | 7 | **0.0** | 0.0 | 0.0 | 0.0 | **5.7** | 2.9 | 8.5 | 69.8 | **43.5** | 22.0 | 65.4 | 70.5 | **683.0** | 683.0 | 683.0 | 0.0 | **755.6** | 719.8 | 791.9 | 6.7 | [[40](#_ENREF_40),[41](#_ENREF_41),[48](#_ENREF_48),[56](#_ENREF_56)] |
| 15 Plantation Forest | 0.10 | **89.4** | 49.9 | 129.1 | 62.6 | 48 | **4.4** | 2.5 | 6.3 | 61.6 | **5.3** | 3.1 | 7.7 | 60.9 | **21.9** | 12.3 | 31.4 | 61.7 | **113.3** | 103.6 | 123.0 | 12.1 | **234.3** | 171.4 | 297.4 | 38.0 | [[16](#_ENREF_16),[21](#_ENREF_21),[45](#_ENREF_45),[57](#_ENREF_57),[58](#_ENREF_58),[59](#_ENREF_59),[60](#_ENREF_60),[61](#_ENREF_61)] |
| 16 Rock outcrops | 0.00 | **2.0** | 2.0 | 4.9 | 151.5 | 6 | **0.6** | 0.6 | 1.5 | 139.5 | **0.8** | 0.8 | 2.0 | 151.5 | **0.0** | 0.0 | 0.0 | 0.0 | **98.4** | 78.1 | 120.2 | 30.3 | **101.8** | 81.5 | 128.7 | 33.1 | [[45](#_ENREF_45),[48](#_ENREF_48),[54](#_ENREF_54)] & unpublished data |
| 17 Urban Area | 0.03 | **0.0** | 0.0 | 0.0 | 0.0 | 0 | **0.0** | 0.0 | 0.0 | 0.0 | **0.0** | 0.0 | 0.0 | 0.0 | **0.0** | 0.0 | 0.0 | 0.0 | **110.1** | 96.8 | 125.2 | 18.3 | **110.1** | 96.8 | 125.2 | 18.3 | [[45](#_ENREF_45)] |
| 18 Water | 0.12 | **0.0** | 0.0 | 0.0 | 0.0 | 0 | **0.0** | 0.0 | 0.0 | 0.0 | **0.0** | 0.0 | 0.0 | 0.0 | **0.0** | 0.0 | 0.0 | 0.0 | **90.2** | 78.9 | 102.0 | 18.1 | **90.2** | 78.9 | 102.0 | 18.1 | [[45](#_ENREF_45)] |
| 19 Woodland with scattered cropland | 2.12 | **48.3** | 28.6 | 64.1 | 52.3 | 7 | **4.5** | 2.8 | 6.0 | 50.6 | **8.1** | 5.0 | 10.7 | 50.2 | **15.4** | 9.1 | 20.3 | 52.0 | **107.0** | 101.9 | 112.3 | 6.9 | **183.3** | 147.4 | 213.5 | 25.6 | [[12](#_ENREF_12),[45](#_ENREF_45),[46](#_ENREF_46),[62](#_ENREF_62)] |
| 20 Lowland Forest | 0.15 | **206.7** | 194.9 | 219.5 | 8.4 | 1228 | **10.1** | 9.5 | 10.8 | 8.7 | **12.2** | 11.5 | 12.9 | 8.6 | **50.4** | 47.5 | 53.6 | 8.6 | **107.1** | 97.6 | 117.5 | 13.1 | **386.5** | 361.0 | 414.3 | 9.8 | [[1](#_ENREF_1),[15](#_ENREF_15),[32](#_ENREF_32),[45](#_ENREF_45)] & unpublished data |
| 21 Sub-montane forest | 0.34 | **283.2** | 252.1 | 328.9 | 19.5 | 184 | **13.9** | 12.4 | 16.1 | 19.2 | **16.7** | 14.8 | 19.4 | 19.8 | **69.1** | 61.5 | 80.1 | 19.4 | **107.4** | 100.0 | 115.4 | 10.1 | **490.2** | 440.8 | 559.9 | 17.4 | [[16](#_ENREF_16),[45](#_ENREF_45),[63](#_ENREF_63),[64](#_ENREF_64)] & unpublished data |
| 22 Montane Forest | 0.21 | **228.3** | 189.6 | 286.1 | 30.5 | 203 | **11.2** | 9.3 | 14.0 | 30.7 | **13.4** | 11.1 | 16.9 | 31.0 | **55.6** | 46.0 | 69.7 | 30.5 | **120.0** | 111.1 | 130.8 | 11.6 | **428.5** | 367.2 | 517.4 | 25.2 | [[45](#_ENREF_45)] & unpublished data |
| 23 Upper-montane forest | 0.10 | **201.7** | 72.7 | 331.7 | 90.8 | 32 | **9.8** | 3.6 | 16.3 | 91.4 | **11.8** | 4.3 | 19.6 | 91.3 | **49.2** | 17.7 | 81.3 | 91.5 | **141.1** | 120.5 | 169.2 | 24.7 | **413.7** | 218.7 | 618.0 | 68.3 | [[45](#_ENREF_45)] & unpublished data |
| 24 Sisal plantation | 0.01 | **54.8** | 44.1 | 67.5 | 30.4 | 3 | **1.9** | 1.5 | 2.3 | 30.4 | **4.3** | 3.5 | 5.3 | 30.4 | **14.7** | 11.8 | 18.1 | 30.4 | **105.6** | 85.4 | 128.1 | 28.6 | **181.3** | 146.3 | 221.3 | 29.3 | [[45](#_ENREF_45),[65](#_ENREF_65)] |
| 25 Tea plantation | 0.02 | **35.1** | 21.9 | 47.6 | 51.9 | 22 | **1.2** | 0.7 | 1.6 | 52.1 | **2.8** | 1.7 | 3.8 | 52.2 | **9.3** | 5.8 | 12.7 | 52.0 | **115.5** | 97.7 | 135.4 | 23.1 | **163.8** | 127.9 | 201.2 | 31.6 | [[13](#_ENREF_13),[45](#_ENREF_45),[47](#_ENREF_47)] |
| 26 Rubber plantation | 0.00 | **74.9** | 44.0 | 80.6 | 41.9 | 2 | **3.7** | 2.2 | 3.9 | 41.9 | **4.4** | 2.6 | 4.7 | 41.9 | **18.3** | 10.8 | 19.7 | 41.9 | **82.8** | 60.5 | 113.5 | 45.8 | **184.0** | 120.1 | 222.5 | 40.6 | [[45](#_ENREF_45),[66](#_ENREF_66)] |
| 27 Teak plantation | 0.00 | **31.4** | 18.5 | 46.7 | 63.8 | 29 | **1.5** | 0.9 | 2.3 | 62.7 | **1.9** | 1.1 | 2.7 | 62.8 | **7.7** | 4.6 | 11.5 | 63.6 | **81.7** | 57.5 | 111.4 | 46.9 | **124.2** | 82.5 | 174.6 | 52.6 | [[21](#_ENREF_21),[45](#_ENREF_45),[59](#_ENREF_59)] |
| 28 Rice plantation | 0.06 | **3.0** | 2.9 | 3.6 | 20.5 | 2 | **0.1** | 0.1 | 0.1 | 20.5 | **0.2** | 0.2 | 0.3 | 20.5 | **0.8** | 0.8 | 1.0 | 20.5 | **177.7** | 104.1 | 252.8 | 59.1 | **181.8** | 108.2 | 257.7 | 58.2 | [[45](#_ENREF_45),[67](#_ENREF_67),[68](#_ENREF_68)] |
| 29 Monocrop unspecified | 0.01 | **2.8** | 1.7 | 5.0 | 88.5 | 13 | **0.1** | 0.1 | 0.2 | 88.5 | **0.2** | 0.1 | 0.4 | 88.8 | **0.8** | 0.5 | 1.3 | 87.0 | **107.9** | 91.3 | 127.5 | 23.8 | **111.7** | 93.6 | 134.4 | 26.0 | [[14](#_ENREF_14),[45](#_ENREF_45),[47](#_ENREF_47),[48](#_ENREF_48),[49](#_ENREF_49)] |
| 30 Sugarcane plantation | 0.01 | **46.3** | 41.9 | 52.1 | 15.7 | 21 | **1.6** | 1.4 | 1.8 | 15.8 | **3.7** | 3.3 | 4.1 | 15.9 | **12.4** | 11.2 | 13.9 | 15.7 | **161.9** | 76.8 | 283.9 | 91.9 | **225.7** | 134.7 | 355.8 | 70.3 | [[45](#_ENREF_45),[69](#_ENREF_69)] |
