# Supplementary material for: Towards Regional, Error-Bounded Landscape Carbon Storage Estimates for Data-Deficient Areas of the World
Source: PLoS One. 2012 Sep 14;7(9):e44795. doi: 10.1371/journal.pone.0044795 (PMC3443093; doi:10.1371/journal.pone.0044795)
Supplement: Table S4 — The carbon values and confidence limits for the aboveground live carbon pool using the original and harmonised land cover categories without any form of weighting. These values are not significantly different from the weighted values shown in Table 3 and Table S3 when a paired t-test is performed (p-value<0.693). The range of landscape values derived from these (1.22 [0.91–1.61] Pg C and 1.70 [1.46–1.98] Pg C for original and harmonised land cover categories respectively) overlap those derived from the weighted values and are also significantly higher than previous estimates (Table 2). (Area (million ha); M - Median carbon storage (Mg ha−1); lCI - Lower 95% confidence interval of carbon storage (Mg ha−1); uCI - Upper 95% confidence interval of carbon storage (Mg ha−1); n – Sample size). (DOCX) [file pone.0044795.s006.docx]

**Table S4 –** The carbon values and confidence limits for the aboveground live carbon pool using the original and harmonised land cover categories without any form of weighting. These values are not significantly different from the weighted values shown in Table 3 and Table S3 when a paired t-test is performed (p-value < 0.693). The range of landscape values derived from these (1.22 [0.91-1.61] Pg C and 1.70 [1.46-1.98] Pg C for original and harmonised land cover categories respectively) overlap those derived from the weighted values and are also significantly higher than previous estimates (Table 2). (Area (million ha); M - Median carbon storage (Mg ha^-1^); lCI - Lower 95% confidence interval of carbon storage (Mg ha^-1^); uCI - Upper 95% confidence interval of carbon storage (Mg ha^-1^); n – Sample size)

| Description | Area | Aboveground live | | | | References |
| --- | --- | --- | --- | --- | --- | --- |
|  |  | **M** | lCI | uCI | **n** |  |
| 1 Unclassified | 0.00 | 0.0 | 0.0 | 0.0 | 0 | [[45](#_ENREF_45)] |
| 2 Bare Soils | 0.02 | 0.0 | 0.0 | 0.0 | 1 | [[45](#_ENREF_45)] & unpublished data |
| 3 Bushland | 5.04 | 121.2 | 47.0 | 201.1 | 15 | [[1](#_ENREF_1),[8](#_ENREF_8),[45](#_ENREF_45)] & unpublished data |
| 4 Bushland with scattered cropland | 3.41 | 0.0 | 0.0 | 0.0 | 1 | [[14](#_ENREF_14),[45](#_ENREF_45)] |
| 5 Closed Woodland | 1.82 | 46.9 | 41.9 | 52.1 | 104 | [[32](#_ENREF_32),[34](#_ENREF_34),[45](#_ENREF_45)] & unpublished data |
| 6 Cultivation | 2.46 | 17.4 | 11.1 | 27.1 | 14 | [[14](#_ENREF_14),[45](#_ENREF_45),[46](#_ENREF_46),[47](#_ENREF_47),[48](#_ENREF_48),[49](#_ENREF_49)] |
| 7 Forest mosaic | 0.05 | 48.6 | 32.5 | 67.5 | 573 | [[45](#_ENREF_45)] & unpublished data |
| 8 Grassland | 5.18 | 3.3 | 1.2 | 6.4 | 7 | [[8](#_ENREF_8),[12](#_ENREF_12),[45](#_ENREF_45),[50](#_ENREF_50),[51](#_ENREF_51)] |
| 9 Grassland with scattered cropland | 2.73 | 0.0 | 0.0 | 0.0 | 3 | [[12](#_ENREF_12),[45](#_ENREF_45),[46](#_ENREF_46)] |
| 10 Ice | 0.00 | 0.0 | 0.0 | 0.0 | 0 | [[45](#_ENREF_45)] |
| 11 Mangrove forest | 0.06 | 27.2 | 12.3 | 43.9 | 6 | [[20](#_ENREF_20),[29](#_ENREF_29),[30](#_ENREF_30),[45](#_ENREF_45),[52](#_ENREF_52),[53](#_ENREF_53)] |
| 12 Ocean | 0.00 | 2.0 | 2.0 | 2.0 | 0 | [[45](#_ENREF_45)] |
| 13 Open Woodland | 9.62 | 196.5 | 187.7 | 205.8 | 114 | [[32](#_ENREF_32),[34](#_ENREF_34),[37](#_ENREF_37),[44](#_ENREF_44),[45](#_ENREF_45),[48](#_ENREF_48),[54](#_ENREF_54),[55](#_ENREF_55)] & unpublished data |
| 14 Permanent Swamp | 0.18 | 289.9 | 262.1 | 319.4 | 7 | [[40](#_ENREF_40),[41](#_ENREF_41),[48](#_ENREF_48),[56](#_ENREF_56)] |
| 15 Plantation Forest | 0.10 | 253.3 | 198.7 | 325.5 | 48 | [[16](#_ENREF_16),[21](#_ENREF_21),[45](#_ENREF_45),[57](#_ENREF_57),[58](#_ENREF_58),[59](#_ENREF_59),[60](#_ENREF_60),[61](#_ENREF_61)] |
| 16 Rock outcrops | 0.00 | 103.9 | 74.4 | 143.5 | 6 | [[45](#_ENREF_45),[48](#_ENREF_48),[54](#_ENREF_54)] & unpublished data |
| 17 Urban Area | 0.03 | 45.7 | 36.8 | 56.4 | 0 | [[45](#_ENREF_45)] |
| 18 Water | 0.12 | 22.2 | 16.0 | 30.4 | 0 | [[45](#_ENREF_45)] |
| 19 Woodland with scattered cropland | 2.12 | 59.0 | 41.7 | 76.3 | 7 | [[12](#_ENREF_12),[45](#_ENREF_45),[46](#_ENREF_46),[62](#_ENREF_62)] |
| 20 Lowland Forest | 0.15 | 31.8 | 18.7 | 47.2 | 1228 | [[1](#_ENREF_1),[15](#_ENREF_15),[32](#_ENREF_32),[45](#_ENREF_45)] & unpublished data |
| 21 Sub-montane forest | 0.34 | 2.7 | 2.5 | 3.0 | 184 | [[16](#_ENREF_16),[45](#_ENREF_45),[63](#_ENREF_63),[64](#_ENREF_64)] & unpublished data |
| 22 Montane Forest | 0.21 | 19.0 | 5.9 | 34.7 | 203 | [[45](#_ENREF_45)] & unpublished data |
| 23 Upper-montane forest | 0.10 | 51.7 | 26.0 | 88.1 | 32 | [[45](#_ENREF_45)] & unpublished data |
| 24 Sisal plantation | 0.01 | 32.3 | 24.3 | 40.0 | 3 | [[45](#_ENREF_45),[65](#_ENREF_65)] |
| 25 Tea plantation | 0.02 | 6.8 | 6.8 | 6.8 | 22 | [[13](#_ENREF_13),[45](#_ENREF_45),[47](#_ENREF_47)] |
| 26 Rubber plantation | 0.00 | 64.5 | 56.5 | 73.6 | 2 | [[45](#_ENREF_45),[66](#_ENREF_66)] |
| 27 Teak plantation | 0.00 | 18.0 | 6.0 | 32.9 | 29 | [[21](#_ENREF_21),[45](#_ENREF_45),[59](#_ENREF_59)] |
| 28 Rice plantation | 0.06 | 176.2 | 163.7 | 189.3 | 2 | [[45](#_ENREF_45),[67](#_ENREF_67),[68](#_ENREF_68)] |
| 29 Monocrop unspecified | 0.01 | 4.1 | 1.2 | 8.6 | 13 | [[14](#_ENREF_14),[45](#_ENREF_45),[47](#_ENREF_47),[48](#_ENREF_48),[49](#_ENREF_49)] |
| 30 Sugarcane plantation | 0.01 | 8.0 | 6.0 | 11.0 | 21 | [[45](#_ENREF_45),[69](#_ENREF_69)] |
| Forest | 0.96 | 207.2 | 197.0 | 218.7 | 1703 | [[1](#_ENREF_1),[15](#_ENREF_15),[16](#_ENREF_16),[20](#_ENREF_20),[21](#_ENREF_21),[29](#_ENREF_29),[30](#_ENREF_30),[32](#_ENREF_32),[45](#_ENREF_45),[52](#_ENREF_52),[53](#_ENREF_53),[57](#_ENREF_57),[58](#_ENREF_58),[59](#_ENREF_59),[60](#_ENREF_60),[61](#_ENREF_61),[63](#_ENREF_63),[64](#_ENREF_64),[66](#_ENREF_66)] & unpublished data |
| Savanna spectrum | 26.02 | 53.1 | 47.3 | 59.8 | 185 | [[1](#_ENREF_1),[8](#_ENREF_8),[12](#_ENREF_12),[32](#_ENREF_32),[34](#_ENREF_34),[37](#_ENREF_37),[40](#_ENREF_40),[41](#_ENREF_41),[44](#_ENREF_44),[45](#_ENREF_45),[48](#_ENREF_48),[50](#_ENREF_50),[51](#_ENREF_51),[54](#_ENREF_54),[55](#_ENREF_55),[56](#_ENREF_56),[70](#_ENREF_70),[71](#_ENREF_71),[72](#_ENREF_72)] & unpublished data |
| Crop | 6.69 | 18.1 | 5.9 | 32.5 | 14 | [[14](#_ENREF_14),[45](#_ENREF_45),[46](#_ENREF_46),[47](#_ENREF_47),[48](#_ENREF_48),[49](#_ENREF_49)] |
| Other | 0.19 | 3.2 | 1.2 | 6.5 | 6 | [[45](#_ENREF_45),[48](#_ENREF_48),[54](#_ENREF_54)] & unpublished data |
