# Supplementary material for: Towards Regional, Error-Bounded Landscape Carbon Storage Estimates for Data-Deficient Areas of the World
Source: PLoS One. 2012 Sep 14;7(9):e44795. doi: 10.1371/journal.pone.0044795 (PMC3443093; doi:10.1371/journal.pone.0044795)
Supplement: References S1 — Citations for the data included in Tables S2, S3, S4. (DOCX) [file pone.0044795.s007.docx]

**References S1 –** Citations for the data included in Table S2-4.

1. Glenday J (2008) Carbon storage and emissions offset potential in an African dry forest, the Arabuko-Sokoke Forest, Kenya. Environmental Monitoring and Assessment 142: 85-95.

2. Isango JA (2006) Monitoring Growth and Impact of Harvesting Options, Shifting Cultivation and Grazing on Vegetation Growth in Miombo Woodlands of Iringa District, Tanzania. Morogoro, Tanzania.: Tanzania Forestry Research Institute.

3. Backéus I, Pettersson B, Strömquist L, Ruffo C (2006) Tree communities and structural dynamics in miombo (Brachystegia-Julbernardia) woodland, Tanzania. Forest Ecology and Management 230: 171-178.

4. Eaton JM, Lawrence D (2009) Loss of carbon sequestration potential after several decades of shifting cultivation in the Southern Yucatán. Forest Ecology and Management 258: 949-958.

5. Mills AJ, Cowling RM, Fey MV, Kerley GIH, Donaldson JS, et al. (2005) Effects of goat pastoralism on ecosystem carbon storage in semiarid thicket, Eastern Cape, South Africa. Carlton, Australia: Blackwell. 8 p.

6. Werf GRVD, Randerson JT, Collatz GJ, Giglio L (2003) Carbon emissions from fires in tropical and subtropical ecosystems. Global Change Biology 9: 547-562.

7. Delaney M, Brown S, Lugo AE, Torres-Lezama A, Quintero NB (1998) The Quantity and Turnover of Dead Wood in Permanent Forest Plots in Six Life Zones of Venezuela. Biotropica 30: 2-11.

8. Mills AJ, O'Connor TG, Donaldson JS, Fey MV, Skowno AL, et al. (2005) Ecosystem carbon storgae under different land uses in three semi-arid shrublands and a mesic grassland in South Africa. South African Journal of Plant and Soil 22: 183-190.

9. Manlay RJ, Ickowicz A, Masse D, Floret C, Richard D, et al. (2004) Spatial carbon, nitrogen and phosphorus budget of a village in the West African savanna--I. Element pools and structure of a mixed-farming system. Agricultural Systems 79: 55-81.

10. Mortimore M, Harris FMA, Turner B (1999) Implications of Land Use Change for the Production of Plant Biomass in Densely Populated Sahelo-Sudanian Shrub-Grasslands in North-East Nigeria. Global Ecology and Biogeography 8: 243-256.

11. Tschakert P, Coomes OT, Potvin C (2007) Indigenous livelihoods, slash-and-burn agriculture, and carbon stocks in Eastern Panama. Ecological Economics 60: 807-820.

12. Manlay RJ, Kairé M, Masse D, Chotte J-L, Ciornei G, et al. (2002) Carbon, nitrogen and phosphorus allocation in agro-ecosystems of a West African savanna I. The plant component under semi-permanent cultivation. Agriculture, Ecosystems and Environment 88: 215-232.

13. Ng'etich WK, Stephens W (2001) Responses Of Tea To Environment In Kenya. 2. Dry Matter Production And Partitioning. Experimental Agriculture 37: 343-360.

14. Schroth G, Zech W (1995) Above- and below-ground biomass dynamics in a sole cropping and an alley cropping system with *Gliricidia sepium* in the semi-deciduous rainforest zone of West Africa. Agroforestry Systems 31: 181-198.

15. Lewis SL, Lopez-Gonzalez G, Sonke B, Affum-Baffoe K, Baker TR, et al. (2009) Increasing carbon storage in intact African tropical forests. Nature 457: 1003-1006.

16. Glenday J (2006) Carbon storage and emissions offset potential in an East African tropical rainforest. Forest Ecology and Management 235: 72-83.

17. Sierra CA, del Valle JI, Orrego SA, Moreno FH, Harmon ME, et al. (2007) Total carbon stocks in a tropical forest landscape of the Porce region, Colombia. Forest Ecology and Management 243: 299-309.

18. Malhi Y, Aragão LEOC, Metcalfe DB, Paiva R, Quesada CA, et al. (2009) Comprehensive assessment of carbon productivity, allocation and storage in three Amazonian forests. Global Change Biology 15: 1255-1274.

19. Cairns MA, Brown S, Helmer EH, Baumgardner GA (1997) Root biomass allocation in the world's upland forests. Oecologia 111: 1-11.

20. Kairo JG, K.S.Lang’at J, Dahdouh-Guebas F, Bosire J, Karachi M (2008) Structural development and productivity of replanted mangrove plantations in Kenya. Forest Ecology and Management 255: 2670-2677.

21. Kraenzel M, Castillo A, Moore T, Potvin C (2002) Carbon storage of harvest-age teak (Tectona grandis) plantations, Panama. Forest Ecology and Management 5863: 1-13.

22. Murphy PG, Lugo AE (1986) Ecology of Tropical Dry Forest. Annual Review of Ecology and Systematics 17: 67-88.

23. IPCC (2006) IPCC Guidelines for National Greenhouse Gas Inventories. Volume 4: Agriculture, Forestry and Other Land Use.

24. Golley FB, McGinnis JT, Clements RG, Child GI, Duever MJ (1969) The Structure of Tropical Forests in Panama and Colombia. BioScience 19: 693-696.

25. Matta SB (1997) Species and structural composition of natural mangrove forests and the implications on management practices: a case study of the Rufiji Delta, Tanzania. Disertation: Sokoine University of Agriculture.

26. Diop ES, Gordon C, Semesi AK, Soumare A, Diallo N, et al. (2002) Mangroves of Africa; Lacerda LDd, editor. New York: Springer-Verlag Berlin Heidelberg.

27. Lugo AE, Snedaker SC (1974) The Ecology of Mangroves. Annual Review of Ecology and Systematics 5: 39-64.

28. Christensen B (1978) Biomass and primary production of Rhizophora apiculata Bl. in a mangrove in southern Thailand. Aquatic Botany 4: 43-52.

29. Steinke TD, Ward CJ, Rajh A (1995) Forest structure and biomass of mangroves in the Mgeni estuary, South Africa. Hydrobiologia 295: 159-166.

30. Twilley RR, Chen RH, Hargis T (1992) Carbon sinks in mangroves and their implications to carbon budget of tropical coastal ecosystems. Water, Air, & Soil Pollution 64: 265-288.

31. Friedel M (1981) Studies of Central Australian Semidesert Rangelands. I. Range Condition and the Biomass Dynamics of the Herbage Layer and Litter. Australian Journal of Botany 29: 219-231.

32. Michelsen A, Andersson M, Jensen M, Kjøller A, Gashew M (2004) Carbon stocks, soil respiration and microbial biomass in fire-prone tropical grassland, woodland and forest ecosystems. Soil Biology & Biochemistry 36: 1707-1717.

33. Bullock SH, editor (1995) Seasonally Dry Tropical Forests. Cambridge: Cambridge University Press. 277-303 p.

34. Woomer PL (1993) The impact of cultivation on carbon fluxes in woody savannas of southern Africa. Water, Air, and Soil Pollution 70: 403-412.

35. De Castro EA, Kauffman JB (1998) Ecosystem structure in the Brazilian Cerrado: a vegetation gradient of aboveground biomass, root mass and consumption by fire. Journal of Tropical Ecology 14: 263-283.

36. Savadogo P (2007) Dynamics of Sudanian savanna-woodland ecosystem in response to disturbances: Acta Universitatis agriculturae Sueciae.

37. Rutherford MC (1993) Empiricism and the prediction of primary production at the mesoscale: A savanna example. Ecological Modelling 67: 129-146.

38. Thompson K (1976) Swamp development in the headwaters of the White Nile; Rzoska J, editor. The Hague, Netherlands: Junk.

39. Thompson K, Shewry PR, Woolhouse HW (1979) Papyrus swamp development in the Upemba Basin, Za&iuml;re: studies of population structure in *Cyperus papyrus* stands. Botanical Journal of the Linnean Society 78: 299-316.

40. Jones MB, Muthuri FM (1997) Standing Biomass and Carbon Distribution in a Papyrus (Cyperus papyrus L.) Swamp on Lake Naivasha Kenya. Journal of Tropical Ecology 13: 347-356.

41. Saunders MJ, Jones MB, Kansiime F (2007) Carbon and water cycles in tropical papyrus wetlands. Wetlands Ecology and Management 15: 489-498.

42. Boaler SB, Sciwale KC (1966) Ecology of a Miombo Site, Lupa North Forest Reserve, Tanzania: III. Effects on the Vegetation of Local Cultivation Practices. Journal of Ecology 54: 577-587.

43. Harmon ME, Whigham DF, Sexton J, Olmsted I (1995) Decomposition and Mass of Woody Detritus in the Dry Tropical Forests of the Northeastern Yucatan Peninsula, Mexico. Biotropica 27: 305-316.

44. Tothill JC, Mott JC (1985) Second International Savannah Symposium, CSIRO, Brisbane, Queensland, Australia. Published in The World’s Savannah: Ecology and Management: Australian Academy of Science. University of Queensland Press.

45. Batjes NH (2004) SOTER-based soil parameter estimates for Southern Africa. Wageningen: ISRIC - World Soil Information. 27 p.

46. Lal R, Kimble JM, Follett RF, Stewart BA, editors (2001) Advances in Soil Science: Assessment Methods for Soil Carbon: Lewis Publishers.

47. Kamau D, Spiertz J, Oenema O (2008) Carbon and nutrient stocks of tea plantations differing in age, genotype and plant population density. Plant and Soil 307: 29-39.

48. Prentice IC (2001) The Carbon Cycle and Atmospheric Carbon Dioxide. Climate Change 2001: The Scientific Basis Cambridge, UK: IPCC, Cambridge University Press.

49. Stoorvogel JJ, Smaling EMA, Janssen BH (1993) Calculating soil nutrient balances in Africa at different scales: I. Supra-national scale. Fertilizer Research 35: 227-235.

50. Deshmukh I (1986) Primary Production of a Grassland in Nairobi National Park, Kenya Journal of Applied Ecology 23: 115-123.

51. Hartemink A (2004) Nutrient stocks of short-term fallows on a high base status soil in the humid tropics of Papua New Guinea. Agroforestry Systems 63: 33-43.

52. de Boer WF (2000) Biomass dynamics of seagrasses and the role of mangrove and seagrass vegetation as different nutrient sources for an intertidal ecosystem. Aquatic Botany 66: 225-239.

53. Slim F, Gwada P, Kodjo M, Hemminga M (1996) Biomass and litterfall of *Ceriops tagal* and *Rhizophora mucronata* in the mangrove forest of Gazi Bay, Kenya. Marine and Freshwater Research 47: 999-1007.

54. Lioubimtseva E, Simon B, Faure H, Faure-Denard L, Adams JM (1998) Impacts of climatic change on carbon storage in the Sahara-Gobi desert belt since the Last Glacial Maximum. Global and Planetary Change 16-17: 95-105.

55. Scholes RJ, Walker BH (1993) An African savannas: synthesis of the Nylsvley study. Cambridge, UK.: Cambridge University Press.

56. Lal R (2005) Forest soils and carbon sequestration. Forest Ecology and Management 220: 242-258.

57. Chamshama SAO, Philip M (1980) Thinning Pinus patula plantations at Sao Hill, Southern Tanzania. Record No. 13. Division of Forestry, Faculty of Agriculture, Forestry and Veterinary Science, University of Dar es Salaam. 16 p.

58. Kaonga ML (2005) Understanding carbon dynamics in agroforestry systems in Eastern Zambia. PhD Thesis: Fitzwilliam College, University of Cambridge.

59. Nunifu TK (1997) The growth and yield of Teak (Tectona grandis Linn F.) - Plantations in Northern Ghana. Masters theisis, Faculty of Forestry, Lakehead University, Thunder Bay, Ontario.

60. Schroeder P (1994) Carbon storage benefits of agroforestry systems. Agroforestry Systems 27: 89-97.

61. Unruh J.D., Houghton R.A., Lefebvre PA (1993) Carbon storgae in agroforesty: an estimate for sub-Saharan Africa. Climate Research 3: 39-52.

62. Nyadzi GI, Otsyina RM, Banzi FM, Bakengesa SS, Gama BM, et al. (2003) Rotational woodlot technology in northwestern Tanzania: Tree species and crop performance. Agroforestry Systems 59: 253-263.

63. Munishi PKT, Shear TH (2004) Carbon storage in afromontane rain forests of the Eastern Arc Mountains of Tanzania: Their net contribution to atmospheric carbon. Journal of Tropical Forest Science 16: 78-93.

64. Zahabu E (2006) Handei Village Forest reserve, Tanzania. Center for International Forestry Research. 16-19 p.

65. Hartemink AE, Wienk JF (1995) Sisal production and soil fertility decline in Tanzania. Outlook on Agriculture 24: 91-96.

66. Wauters JB, Coudert S, Grallien E, Jonard M, Ponette Q (2008) Carbon stock in rubber tree plantations in Western Ghana and Mato Grosso (Brazil). Forest Ecology and Management 255: 2347-2361.

67. Masija EH (1993) Irrigation of wetlands in Tanzania. Morogoro, Tanzania, 27-29 November 1991 IUCN Wetlands Programme.

68. Meertens HCC, Ndege LJ, Lupeja PM (1999) The cultivation of rainfed, lowland rice in Sukumaland, Tanzania. Agriculture, Ecosystems and Environment 76: 31-45.

69. Tarimo AJP, Takamura YT (1998) Sugarcane production, processing and marketing in Tanzania. African Study Monographs 19: 1-11.

70. Ek TM (1994) Biomass structure in miombo woodland and semievergreen forest. MSc Thesis: Agricultural University of Norway. 53 p.

71. Stromgaard P (1985) Biomass, growth, and burning of wood- land in a shifting cultivation area of South Central Africa. Forest Ecology and Management 12: 163-178.

72. Zahabu E (2006) Kitulangalo Forest Area, Tanzania. Center for International Forestry Research. 20-25 p.
